# Effects of bone morphogenetic protein-2 (BMP-2) and vascular endothelial growth factor (VEGF) release from polylactide-poly (ethylene glycol)-polylactide (PELA) microcapsule-based scaffolds on bone

**DOI:** 10.1590/1414-431X20176520

**Published:** 2017-11-30

**Authors:** Q. Ren, M. Cai, K. Zhang, W. Ren, Z. Su, T. Yang, T. Sun, J. Wang

**Affiliations:** 1 Emergency Department, Second Affiliated Hospital of Inner Mongolia Medical University, Hohhot, Inner Mongolia, China; 2 Department of Obstetrics and Gynecology, First Affiliated Hospital of Baotou Medical College, Baotou, Inner Mongolia, China; 3 Department of Cardiothoracic Surgery, Third Affiliated Hospital of Inner Mongolia Medical University, Baotou, Inner Mongolia, China

**Keywords:** Bone tissue engineering, BMP-2, VEGF, PELA, Scaffold

## Abstract

Multiple growth factors can be administered to mimic the natural process of bone healing in bone tissue engineering. We investigated the effects of sequential release of bone morphogenetic protein-2 (BMP-2) and vascular endothelial growth factor (VEGF) from polylactide-poly (ethylene glycol)-polylactide (PELA) microcapsule-based scaffolds on bone regeneration. To improve the double emulsion/solvent evaporation technique, VEGF was encapsulated in PELA microcapsules, to which BMP-2 was attached. The scaffold (BMP-2/PELA/VEGF) was then fused to these microcapsules using the dichloromethane vapor method. The bioactivity of the released BMP-2 and VEGF was then quantified in rat mesenchymal stem cells (rMSCs). Immunoblotting analysis showed that BMP-2/PELA/VEG promoted the differentiation of rMSCs into osteoblasts via the MAPK and Wnt pathways. Osteoblast differentiation was assessed through alkaline phosphatase expression. When compared with simple BMP-2 plus VEGF group and pure PELA group, osteoblast differentiation in BMP-2/PELA/VEGF group significantly increased. An MTT assay indicated that BMP-2-loaded PELA scaffolds had no adverse effects on cell activity. BMP-2/PELA/VEG promoted the differentiation of rMSCs into osteoblast via the ERK1/2 and Wnt pathways. Our findings indicate that the sequential release of BMP-2 and VEGF from PELA microcapsule-based scaffolds is a promising approach for the treatment of bone defects.

## Introduction

Bone tissue engineering aims to generate functional bone tissue for the replacement of defective bone, thus re-establishing normal function in humans (1). Mesenchymal stem cells (MSCs), which can differentiate *in vitro* into various mesenchymal lineages, are widely used for this purpose because they can be easily isolated from different sources (2,3). Bone marrow-derived mesenchymal stem cells (bMSCs) are multipotent adult stem cells, whose osteogenic differentiation potential has been reported in several *in vitro* studies, have become an important source of cells for engineered tissue repair and cell therapy (4). Furthermore, studies in small animal models have shown biodegradable scaffolding implants to significantly improve bone formation, indicating its great potential for therapeutic applications (5).

It has been reported that the degree of microcapsule expression is dependent on the composition of the growth medium, the stage of growth, and whether the organisms are cultured on solid or liquid medium (6,7). For example, growth medium with added VEGF has been found to enhance microcapsule expression, which is attributed to the low-phosphate nature of this medium (8,9).

Vascular endothelial growth factor (VEGF) is a growth factor that promotes epithelial cell proliferation and chemotaxis (10). It has also been shown to regulate bone formation, development and regeneration (11). VEGF has recently been shown to prevent or treat ischemia (1). We hypothesized that rMSC (rat MSC) vascularization in tissue-engineered bone might be improved in the existence of VEGF. Bone morphogenetic proteins (BMPs) are bone growth factors that promote osteogenesis (12,13). Under certain conditions, BMPs can also induce the transformation of undifferentiated mesenchymal cells into bone cells and induce the proliferation of bone cells, indicating that they are one of the most important factors in osteogenesis (14,15). The effects of BMP-2 on the osteogenic differentiation of bMSCs have been reported (14). The bone growth factors that are known to induce osteogenesis and BMP-2 are currently being used in various animal experiments and clinical settings (13–16). In this study, we investigated the effects of BMP-2- and VEGF-based microcapsules on the growth of rMSCs. The involvement of MAPK signaling and Wnt and β-catenin in this process were also explored.

## Material and Methods

### Material

BMP-2 and VEGF were purchased from Sigma (USA). Antibodies to IgG, β-actin, alkaline phosphatase (ALP), EKR1/2, JNK, p38, p-ERK1/2, p-JNK, p-p38, Wnt andβ-catenin were purchased from Cell Signaling Technology (USA).

### Preparation of BMP- and VEGF-loaded microcapsules

Microcapsules containing BMP-2 and VEGF were prepared using the improved double emulsion/solvent evaporation technique as previously described (17). In brief, 3 μg of BMP-2 was dissolved in 200 µL of distilled water, which was then combined with 4 mL of dichloromethane containing 280 mg of polylactide-poly (ethylene glycol)-polylactide (PELA) (MW 20,000). After sonication for 20 min, the primary emulsion was combined with 40 mL of 0.8% polyvinyl alcohol and stirred for 40 min. The microparticles were washed, centrifuged thrice, mixed with 2 mL of phosphate buffered saline (PBS), pH 7.4, containing 3 μg of VEGF and stirred for 10 min. BMP-2-encapsulated microparticles encased by VEGF (BMP-2/PELA/VEGF) were lyophilized overnight and then collected. The four types of microcapsules (groups A, B, C, D and E) are listed in Table 1.

**Table 1.** Microcapsules containing BMP-2 for scaffolds fusing.

Microcapsules	Material	Encapsulated protein	Covered protein
Group A	280 mg PELA	3 μg BMP-2	3 μg VEGF
Group B	280 mg PELA	3 μg BMP-2	–
Group C	280 mg PELA	–	3 μg VEGF
Group D	280 mg PELA	–	–

BMP-2: bone morphogenetic protein-2; PELA: polylactide-poly (ethylene glycol)-polylactide; VEGF: vascular endothelial growth factor.

### Scaffold construction

Microcapsules (polylactic acid-polyethylene glycol-polylactic acid, PLA-PEG-PLA, PELA) were fused with scaffolds using the dichloromethane vapor method as previously described (17). In brief, 30 mg of microcapsules was placed into a 35-mm dish, which was then sealed in a 60-mm dish containing 5 mL of dichloromethane. The scaffolds were incubated for 10 min. After air-drying for 10 min, the scaffolds were sterilized by ethylene oxide and stored at −20°C until further use. Four types of scaffolds were prepared with the indicated microcapsules.

### *In vitro* swelling and degradation of scaffolds

Scaffold swelling and degradation tests were performed in PBS pH 7.4 at 37°C. Sixty milligrams of scaffolds were placed into 15-mL tubes containing 10 mL of PBS and incubated at 37°C. The PBS was replaced every third day. At each time (1, 2, 5, 8, 11, 14, 18, 22, 26, 32, 39, and 46 days), scaffolds were centrifuged and their wet weight (Ww) was recorded. Thereafter, scaffolds were lyophilized for 12 h and their dry weight (Wd) was recorded. The weight loss of each scaffold was calculated. The swelling ratio was calculated as the *in vitro* swelling and degradation.

### BMP-2 and VEGF assays

The release of BMP2 from BMP-2/PELA/VEGF scaffolds in PBS pH 7.4 was measured at 37°C. The concentrations of BMP-2 and VEGF at each time point (1, 2, 4, 8, 12, 16, 22, 28, 35, and 42 days) were measured using human BMP-2 and VEGF. ELISA kits were purchased from R&D Systems, Germany. VEGF and BMP-2 expression was evaluated in an ELISA plate reader at 450 nm with a correction at 570 nm. Results were normalized to picogram VEGF and BMP-2 per hour treatment per 10^4^ cells. This experiment was carried out in triplicate.

### Isolation and culture of rMSCs

rMSCs were obtained from a neonatal New Zealand white rabbit. The bone marrow was flushed with a 1-mL syringe containing low glucose Dulbecco's modified Eagle's medium (DMEM). Cells were harvested, transferred to a dish, and cultured in an incubator at 37°C with 5% CO_2_. The Inner Mongolia Medical University Experimental Animal Management Committee approved the animal protocol.

### Western blotting

Proteins derived from rMSCs were separated by sodium dodecyl sulfate-polyacrylamide gel electrophoresis using 12% gels and transferred to nitrocellulose membranes for immunoblotting analysis. Membranes were blocked and incubated with primary antibodies overnight at 4°C. Membranes were subsequently washed three times with PBS and incubated with peroxidase-conjugated secondary antibodies. Immunoreactive bands were detected with Pierce ECL reagents (USA). The endogenous control was GAPHD.

### Osteogenic differentiation

rMSCs were seeded on 48-wellplates at 5×10^4^ cell/cm^2^ and incubated with10 mg of scaffolds in every well. The cell culture medium (containing transforming growth factor beta 1 (TGF-β1, 10 mg/mL) was refreshed once every 3 days. Cells were cultured at 37°C in a humidified atmosphere with 5% CO_2_. At days 3, 7, and 14, cells were obtained. Osteogenic differentiation was achieved following established *in vitro* protocols.

### Statistical analyses

Statistical analyses were conducted by one-way analysis of variance (ANOVA) using the GraphPad Prism Software (version 5.0; GraphPad Software, USA, www.graphpad.com/company/). Data are reported as means±SD.

## Results

### Swelling ratio and degradation of scaffolds and the measurement of released BMP-2 and VEGF

**Figure 1.**
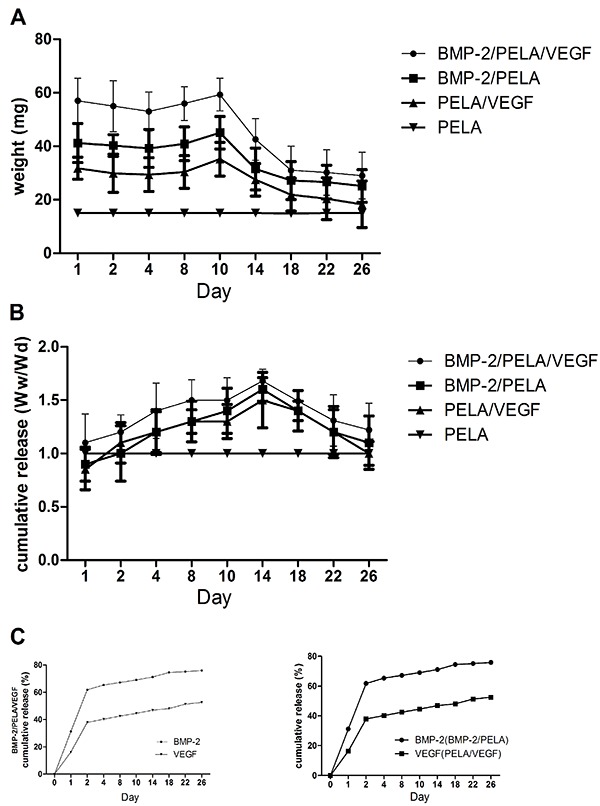
Weight loss (*A*), swelling ratio (*B*) and cumulative release profile (*C*) of BMP-2/PELA/VEGF scaffolds in PBS at 37°C. Data are reported as means±SD. BMP-2: bone morphogenetic protein-2; PELA: polylactide-poly (ethylene glycol)-polylactide; VEGF: vascular endothelial growth factor; Ww: wet weight; Wd: dry weight.

The swelling and degradation of scaffolds in PBS were assessed at 37°C for 28 days. The swelling ratio was significantly higher from days 1 to 14 than from days 15 to 28, reaching its peak on day 14. The swelling ratio then decreased, reaching its lowest level on day 28. Furthermore, there was no significant loss in scaffold weight from days 1 to 12 (Figure 1A), consistent with the increased swelling ratio. However, the scaffold weight decreased from days 13 to 28, and by day 22, the weight was 50% of the initial value.

The concentrations of BMP-2 and VEGF released from BMP-2/PELA/VEGF scaffolds were measured by ELISA (Figure 1C). The BMP-2 level increased by 60% on day 2, followed by a further increase until the end of the experiment. By contrast, the VEGF level increased by only 32% on day 2, followed by an increase of 1% daily. After 3 weeks, the VEGF level reached a plateau.

### Viability of rMSCs in the presence of PELA scaffolds

**Figure 2.**
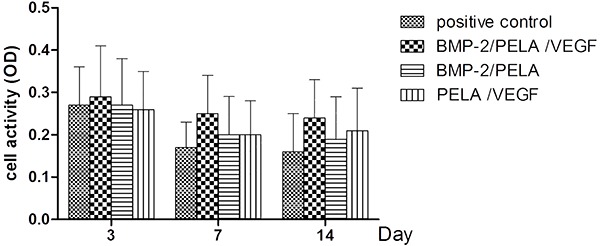
Results of the MTT assay after incubation of cells with PELA scaffolds. Cells cultured in the absence of scaffolds served as the positive control. Data are reported as means±SD. BMP-2: bone morphogenetic protein-2; PELA: polylactide-poly (ethylene glycol)-polylactide; VEGF: vascular endothelial growth factor.

There was no difference in cell viability between PELA and control scaffolds at all time points (Figure 2). Viability was higher at 3 days in cells of group B than in those of the control group, although the difference was not statistically significant. These results indicate that BMP-based PELA scaffolds do not affect cell viability within the first 14 days.

### BMP-2/PELA/VEGF promoted the differentiation of rMSCs into osteoblasts via the ERK1/2 and Wnt pathway

**Figure 3.**
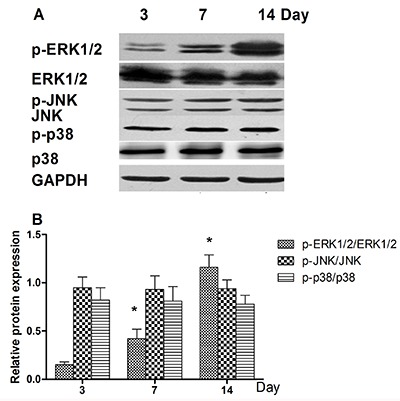
Effects of BMP-2/PELA/VEGF scaffolds on the activation of mitogen-activated protein kinase (MAPK) signaling in rats mesenchymal stem cells. *A*, Expression of total and phosphorylated ERK1/2, JNKs, and p38 proteins. *B*, Contrast gray values corresponding to phosphorylated ERK1/2, JNKs, and p38 based on western blotting analysis. Results are reported as means±SD (n=5). *P<0.05, compared to the 3-day group (ANOVA). BMP-2: bone morphogenetic protein-2; PELA: polylactide-poly (ethylene glycol)-polylactide; VEGF: vascular endothelial growth factor.

**Figure 4.**
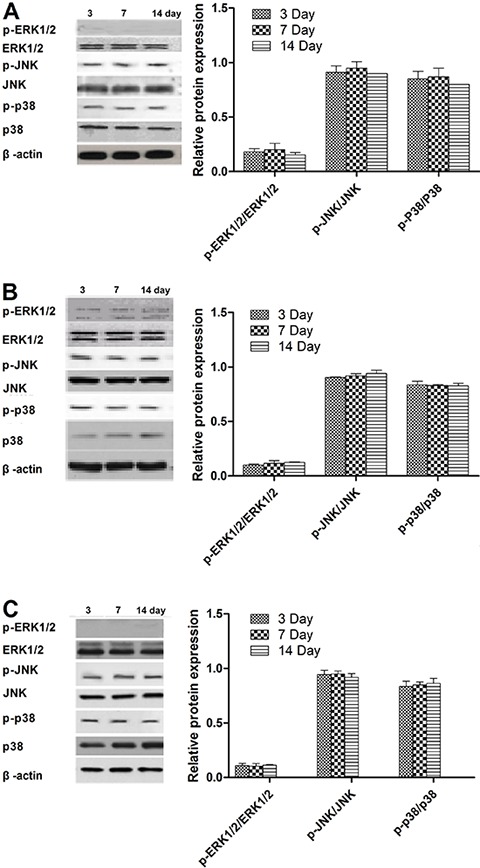
Effects of BMP-2/PELA (*A*) and PELA/VEGF (*B*) scaffolds, as well as the positive control (*C*), on the activation of mitogen-activated protein kinase (MAPK) signaling in rBMSCs cells. Results are reported as means±SD.

To identify the signaling pathway through which BMP-2/PELA/VEG promotes the differentiation of rMSCs into osteoblasts, cells were cultured for 3, 7, and 14 days in the presence of control, BMP-2/PELA/VEGF, BMP-2/PELA or PELA/VEGF scaffolds. The level of phosphorylated ERK1/2 was also examined (Figures 3A and 4). The level of phosphorylated ERK1/2 was higher from days 3 to 14 in cells cultured in the presence of BMP-2/PELA/VEGF scaffolds than in those cultured in the presence of control scaffolds. There were no changes in the levels of phosphorylated JNK and p38 in cells cultured in the presence of BMP-2/PELA/VEGF scaffolds (Figure 3B).

**Figure 5.**
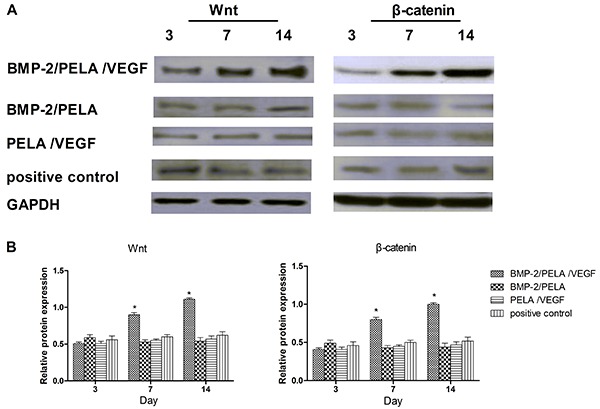
Expression of Wnt and β-catenin proteins in BMP-2/PELA/VEGF and BMP-2/PELA scaffolds, as well as the positive control. *A*, Western blotting images. *B*, Quantification of Wnt and β-catenin expression based on western blotting analysis. Results are reported as means±SD (n=5). *P<0.05, compared to the 3-day group (ANOVA). BMP-2: bone morphogenetic protein-2; PELA: polylactide-poly (ethylene glycol)-polylactide; VEGF: vascular endothelial growth factor.

The Wnt and β-catenin were also detected (Figure 5A and B). The expression level of Wnt and β-catenin were higher from days 3 to 14 in cells cultured in the presence of BMP-2/PELA/VEGF scaffolds than in those cultured in the presence of control scaffolds

**Figure 6.**
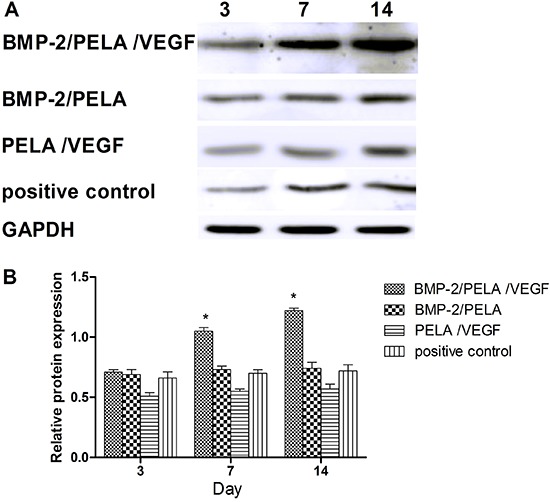
Expression of alkaline phosphatase (ALP) protein in BMP-2/PELA/VEGF and BMP-2/PELA scaffolds, as well as the positive control, 3, 7, and 14 days after mesenchymal stem cells seeding. *A*, Western blotting images. *B*, Quantification of ALP based on western blotting analysis. Results are reported as means±SD (n=5). *P<0.05, compared to the 3-day group (ANOVA). BMP-2: bone morphogenetic protein-2; PELA: polylactide-poly (ethylene glycol)-polylactide; VEGF: vascular endothelial growth factor.

To investigate the effects of BMP-2/PELA/VEGF scaffolds on rMSC-derived osteoblasts, we induced the differentiation of rMSCs into osteoblasts and cultured cells in the presence of control, BMP-2/PELA/VEGF, BMP-2/PELA or PELA/VEGF scaffolds. Thereafter, the ALP level was examined. There was a significant change in the ALP level in cells cultured in the presence of BMP-2/PELA/VEGF scaffolds than in those cultured in the presence of control scaffolds (Figure 6).

## Discussion

Many factors, including porosity, temperature and medium, can affect the scaffold degradation rate (17). For example, triblock polymers are composed of biodegradable PLA and an inlaid hydrophilic PEG block, and PELA has been successfully used as a carrier of both hydrophilic and hydrophobic drugs (8). In this study, PELA was used to construct the microcapsule wall and PBS was used as the degradation medium. Based on the swelling ratio and the weight loss profile, fast degradation was achieved at week 2 after initial swelling, which was similar to previously reported.

In scaffolds, BMP-2 was attached to the surface of PELA microcapsules, which was responsible for its rapid burst release, whereas VEGF was encapsulated by PELA microcapsules, which was responsible for its sustained release. According to the *in vitro* release profile of BMP-2/PELA/VEGF scaffolds, BMP-2 exhibited a classic initial burst release, followed by a sustained release for more than 28 days. This release profile has been reported to improve bone regeneration compared to a sustained release without a burst.

To gain further insight into the mechanism by which BMP-2/PELA/VEGF scaffolds enhance rMSC proliferation, we investigated the involvement of the MAPK signaling pathway. The MAPK signaling pathway is comprised of ERK, JNK and p38 serine/threonine kinases that are primarily involved in the activation of nuclear transcription factors that control cell proliferation, differentiation and apoptosis (18). Extracellular signal-related kinase (ERK) is a member of the MAP kinase family that stimulates the differentiation of hMSC into osteoblasts via phosphorylation of the osteogenic transcription factor runx2/CBFA-1 (19). The Wnt/β-catenin signaling is activated by the binding of Wnt ligands to the frizzled family of receptors (20). The β-catenin signaling also plays an important role in regulating the commitment of the differentiation of pluripotent stem cell into the osteoblasts during fracture healing (21). In this study, the differentiation into osteoblasts represented by ALP activity was promoted by BMP-2/PELA/VEG treatment. Phosphorylation of ERK1/2, Wnt and β-catenin were increased by BMP-2/PELA/VEG treatment. In summary, our results indicate that BMP-2/PELA/VEGF scaffold promoted the differentiation of rMSCs to osteoblasts. These results indicate that BMP-2/PELA/VEGF scaffolds can potentially repair bone defects.

## References

[1] Tan Y, Xiao EH, Xiao LZ, Yuan YH, Ma C, Shang QL (2012). VEGF (165) expressing bone marrow mesenchymal stem cells differentiate into hepatocytes under HGF and EGF induction *in vitro*. Cytotechnology.

[2] Yazdani Y, Sharifi Rad MR, Taghipour M, Chenari N, Ghaderi A, Razmkhah M (2016). Genistein suppression of matrix metalloproteinase 2 (MMP-2) and vascular endothelial growth factor (VEGF) expression in mesenchymal stem cell like cells isolated from high and low grade gliomas. Asian Pac J Cancer Prev.

[3] Liu B, Li X, Liang G, Liu X (2011). VEGF expression in mesenchymal stem cells promotes bone formation of tissue-engineered bones. Mol Med Rep.

[4] Zhou WW, Hu JG, Yang JF, Lin L, Zhou XM, Tang T (2006). [Angiogenic effect of bone marrow mesenchymal stem cells transfected with human VEGF gene on myocardial infarcts in rats]. Zhong Nan Da Xue Xue Bao Yi Xue Ban.

[5] Wang XL, Wang W, Ma J, Guo X, Yu XJ, Qiu ZW (2005). [Microenvironment effect of APA microcapsule on embryonic stem cell]. Sheng Li Xue Bao.

[6] Shigeri Y, Kondo T (1969). Studies on microcapsules. 3. Permeability of polyurethane microcapsule membranes. Chem Pharm Bull.

[7] Sa B, Bandyopadhyay AK, Gupta BK (1996). Effect of microcapsule size and polyisobutylene concentration on the release of theophylline from ethylcellulose microcapsules. J Microencapsul.

[8] Tam SK, de Haan BJ, Faas MM, Halle JP, Yahia L, de Vos P (2009). Adsorption of human immunoglobulin to implantable alginate-poly-L-lysine microcapsules: effect of microcapsule composition. J Biomed Mater Res A.

[9] Benchabane S, Subirade M, Vandenberg GW (2007). Production of BSA-loaded alginate microcapsules: influence of spray dryer parameters on the microcapsule characteristics and BSA release. J Microencapsul.

[10] Wahl EA, Schenck TL, Machens HG, Balmayor ER (2016). VEGF released by deferoxamine preconditioned mesenchymal stem cells seeded on collagen-GAG substrates enhances neovascularization. Sci Rep.

[11] Tomanek RJ, Christensen LP, Simons M, Murakami M, Zheng W, Schatteman GC (2010). Embryonic coronary vasculogenesis and angiogenesis are regulated by interactions between multiple FGFs and VEGF and are influenced by mesenchymal stem cells. Dev Dyn.

[12] Schofer MD, Veltum A, Theisen C, Chen F, Agarwal S, Fuchs-Winkelmann S (2011). Functionalisation of PLLA nanofiber scaffolds using a possible cooperative effect between collagen type I and BMP-2: impact on growth and osteogenic differentiation of human mesenchymal stem cells. J Mater Sci Mater Med.

[13] Vural AC, Odabas S, Korkusuz P, Yar Saglam AS, Bilgic E, Cavusoglu T (2017). Cranial bone regeneration via BMP-2 encoding mesenchymal stem cells. Artif Cells Nanomed Biotechnol.

[14] Hu JJ, Liu YW, He MY, Jin D, Zhao H, Yu B (2014). Proteomic analysis on effectors involved in BMP-2-induced osteogenic differentiation of beagle bone marrow mesenchymal stem cells. Proteome Sci.

[15] Wang CL, Xiao F, Wang CD, Zhu JF, Shen C, Zuo B (2017). Gremlin2 Suppression increases the BMP-2-Induced osteogenesis of human bone marrow-derived mesenchymal stem cells via the BMP-2/Smad/Runx2 signaling pathway. J Cell Biochem.

[16] Wang M, Zou Z (2014). Multiple mechanisms of SDF-1 promoting VEGF-induced endothelial differentiation of mesenchymal stem cells. Int J Cardiol.

[17] Li X, Min S, Zhao X, Lu Z, Jin A (2014). Optimization of entrapping conditions to improve the release of BMP-2 from PELA carriers by response surface methodology. Biomed Mater.

[18] Delhanty PJ, van der Eerden BC, van der Velde M, Gauna C, Pols HA, Jahr H (2006). Ghrelin and unacylated ghrelin stimulate human osteoblast growth via mitogen-activated protein kinase (MAPK)/phosphoinositide 3-kinase (PI3K) pathways in the absence of GHS-R1a. J Endocrinol.

[19] Klees RF, Salasznyk RM, Kingsley K, Williams WA, Boskey A, Plopper GE (2005). Laminin-5 induces osteogenic gene expression in human mesenchymal stem cells through an ERK-dependent pathway. Mol Biol Cell.

[20] Aguilar JS, Begum AN, Alvarez J, Zhang XB, Hong Y, Hao J (2015). Directed cardiomyogenesis of human pluripotent stem cells by modulating Wnt/beta-catenin and BMP signalling with small molecules. Biochem J.

[21] de Boer J, Siddappa R, Gaspa C, van Apeldoorn A, Fodde R, van Blitterswijk C (2004). Wnt signaling inhibits osteogenic differentiation of human mesenchymal stem cells. Bone.

